# Common bile duct exploration with choledochotomy and primary repair during pregnancy: Case Report

**DOI:** 10.3389/fmed.2025.1559568

**Published:** 2025-04-15

**Authors:** Jiaqi Chen, Liyong Zhang, Wenjuan Zhang, Zejin Zhao, Aijun Yu, Jian Li, Zhuqing Zhang, Kai Chen

**Affiliations:** ^1^Department of Hepatobiliary Surgery, The Affiliated Hospital of Chengde Medical University, Chengde, Hebei, China; ^2^Department of Hepatobiliary Surgery, Hebei Key Laboratory of Panvascular Diseases, Affiliated Hospital of Chengde Medical University, Chengde, Hebei, China

**Keywords:** choledocholithiasis, pregnancy, T-tube-free, laparoscopic common bile duct exploration, case report

## Abstract

We present a case of a woman in the second trimester of pregnancy who was admitted due to symptomatic common bile duct stones and gallstones. The patient underwent ultrasonography (US) and magnetic resonance cholangiopancreatography (MRCP) imaging, as well as a series of relevant blood tests, to establish a diagnosis. After a comprehensive assessment, simultaneous T-tube-free laparoscopic transcholedochal stone extraction and cholecystectomy were performed safely in the pregnant patient with common bile duct stones and gallstones. Postoperatively, the patient had an uneventful recovery. This case report aims to provide detailed information on the selection of treatment options for symptomatic choledocholithiasis combined with gallstones during pregnancy and to explore the feasibility and safety of performing concurrent T-tube-free laparoscopic choledochotomy for stone extraction in pregnant patients.

## 1 Introduction

Physiological changes during pregnancy increase the risk of gallstone formation and complicate their management (1). Choledocholithiasis during pregnancy is not common, yet its complications, such as obstructive jaundice, cholangitis, or pancreatitis, pose significant risks to both the mother and the fetus (2). Although there is a consensus on preoperative management and the need to treat cholelithiasis with choledocholithiasis, a debate still exists on how to cure the two diseases at the same time (3). Traditional open cholecystectomy, common bile duct (CBD) exploration, along T-tube drainage are currently not appropriate choices for the majority of patients (4). Over the past two decades, advancements in endoscopic and minimally invasive surgical techniques, coupled with the development of Internet of Things (IoT) technology and the involvement of multidisciplinary management, have led to a diversification of diagnostic and therapeutic options (5, 6). In the treatment of patients with concurrent gallstones and common bile duct stones, common surgical approaches include laparoscopic cholecystectomy (LC) with laparoscopic common bile duct exploration (LCBDE), LC with preoperative endoscopic retrograde cholangiopancreatography (PreERCP), LC with intraoperative endoscopic retrograde cholangiopancreatography (IntraERCP), and LC with postoperative endoscopic retrograde cholangiopancreatography (PostERCP) (7). However, in patients with concurrent gallstones and common bile duct stones during pregnancy, considering the potential teratogenic risks to the fetus from ionizing radiation during ERCP procedures and the threats to the health of pregnant women and fetuses from long-term T-tube placement, it is particularly important to explore a treatment plan that can solely utilize laparoscopic surgery to clear bile duct stones and remove the gallbladder while ensuring the safety of both mother and child. This article reports a case of a young woman in the second trimester of pregnancy who was diagnosed with common bile duct stones and gallstones and underwent a single-session laparoscopic treatment. This study adheres to the SCARE guidelines which are aimed to help further improve the reporting quality of case reports (8). This study aims to explore the feasibility and safety of performing T-tube-free laparoscopic choledochotomy for stone extraction and cholecystectomy in pregnant patients and to provide an in-depth analysis combined with the latest research advancements. The following sections of this paper will provide a detailed account of the clinical presentation and therapeutic course of the case, exploring the technical essentials and benefits of common bile duct exploration with choledochotomy and primary repair during pregnancy, and discuss these aspects in conjunction with relevant literature.

## 2 Case report

**Figure 1:**
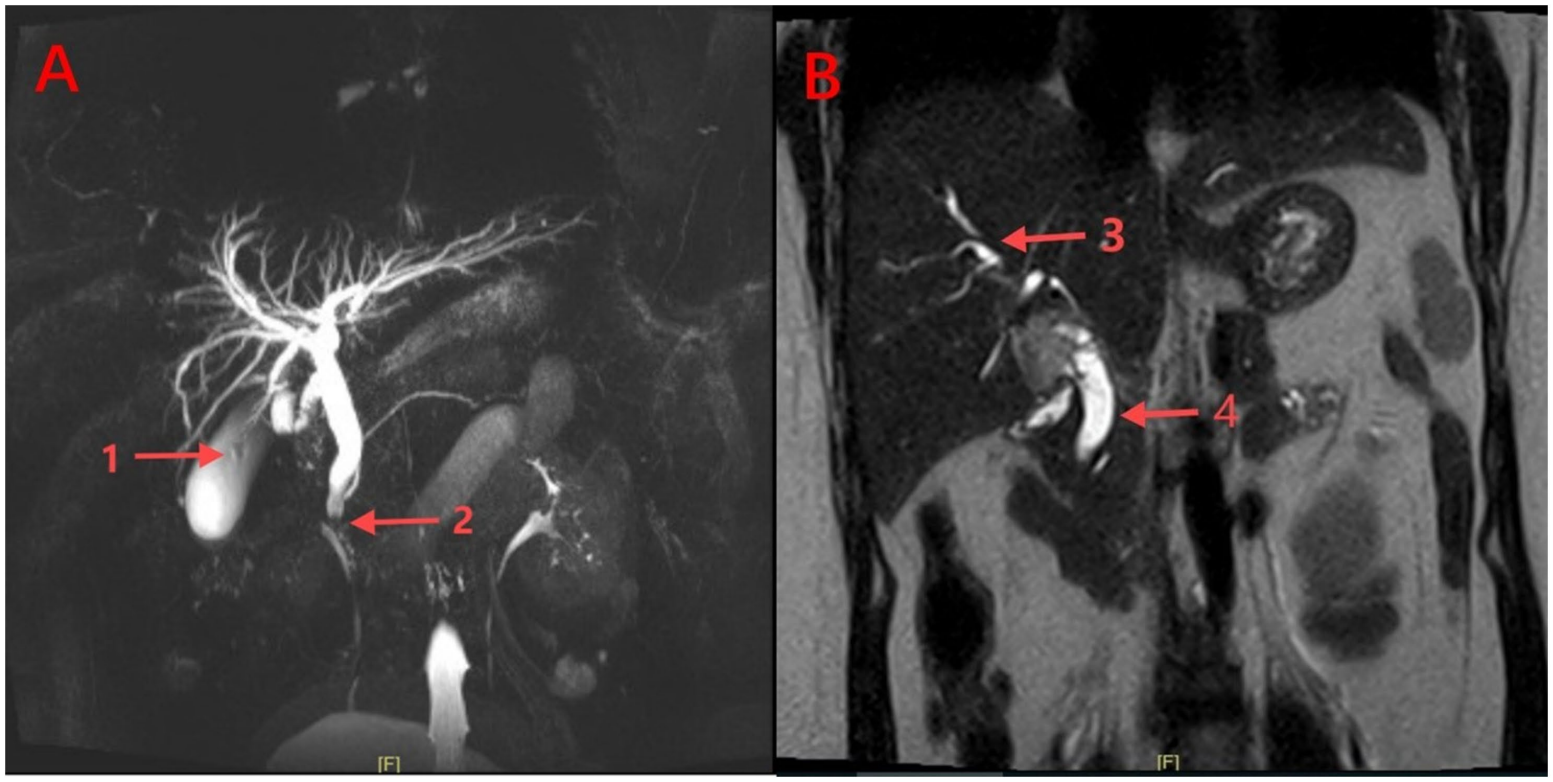
Abdominal MRCP images. **(A)**: (1) Gallstones within the distended gallbladder; (2) Distal common bile duct stones; **(B)**: (3) Dilated intrahepatic bile ducts; (4) Dilated common bile duct (CBD).

A 32-year-old G2P1 woman in the second trimester of pregnancy (18w+) was admitted to the hospital due to right upper quadrant abdominal pain accompanied by skin and scleral icterus for 2 days. The patient reported intermittent pain radiating to the right shoulder and back, associated with nausea and vomiting, darkening of urine, and denied fever, chills, or other changes with a history of cesarean section and no history of heart, lung, or stomach diseases. Vital signs are BP 116/69 mmHg, RR 20 breaths per minute, PR 106 beats per minute, and temperature 37.0°C. Abdominal examination revealed generalized skin and scleral icterus, positive Murphy’s sign, with no signs of peritonitis. The abdominal US reveals an enlarged gallbladder with abnormal echoes within the gallbladder lumen, suggestive of sludge deposition; MRCP reveals a small gallstone and a 3.62 mm diameter stone at the end of the common bile duct (Figure 1A), with dilation of the common bile duct and both intra-and extrahepatic bile ducts, with the common bile duct measuring 11 mm in diameter (Figure 1B). Initial laboratory tests revealed a total bilirubin level of 99.94 μmol/L (normal range 3–22), alanine aminotransferase (ALT) at 133 U/L (normal range 0–35), aspartate aminotransferase (AST) at 65 U/L (normal range 14–36), alkaline phosphatase at 120 U/L (normal range 38–126), serum amylase at 56 U/L (normal range 30–110), and a normal complete blood count (CBC). The preliminary diagnosis is choledocholithiasis, obstructive jaundice, and gallstones with cholecystitis.

After an in-depth discussion by the multidisciplinary team (MDT), and considering the opinions of experts from gastroenterology, anesthesiology, and obstetrics, we decided to avoid endoscopic retrograde cholangiopancreatography (ERCP) to minimize radiation exposure to the fetus. Moreover, given that the patient suffered from obstructive jaundice, there was a possibility of placing a T-tube. In such a case, the approach via the cystic duct might not be appropriate. If the attempt to remove the stones via the cystic duct proved unsuccessful, we would still have to resort to a choledochotomy via the common bile duct. This would not only prolong the operative time and increase the complexity of the procedure but also potentially expose the patient to unnecessary additional risks. After long deliberation, we have resolved to conduct the laparoscopic transcholedochal stone removal and cholecystectomy. Preoperatively, the patient was administered a single intramuscular injection of 20 mg progesterone injection and an intravenous infusion of 40 mL of 25% magnesium sulfate mixed with 500 mL of normal saline, infused at a rate of 1–2 g/h, to suppress uterine contractions. In addition, continuous fetal heart rate monitoring was performed throughout the surgery.

**Figure 2:**
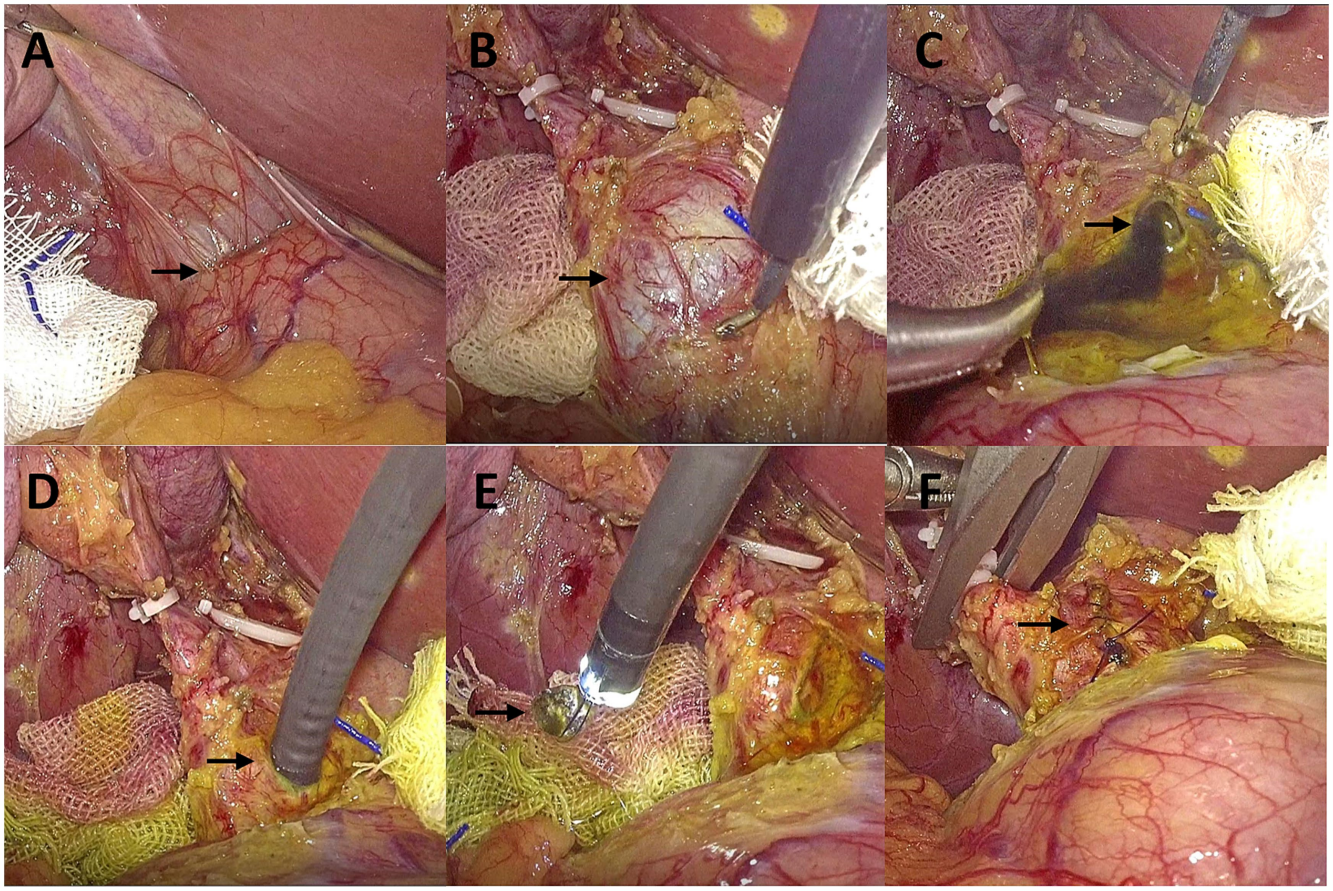
Surgical photographs. **(A)** The cystic duct adheres to the duodenum; **(B)** Common bile duct (CBD) dilation; **(C)** Stagnant bile; **(D)** Insertion of the choledochoscope into the CBD; **(E)** Stone extraction; **(F)** Suture of CBD.

After general endotracheal anesthesia was administered, the patient was positioned in a head-low, foot-high, left-tilted supine position with a pneumoperitoneum pressure maintained at 8–10 mmHg. A standard laparoscopic LC approach was used, with the pneumoperitoneum puncture site located above the umbilicus. A 12-mm trocar was placed below the xiphoid process and a 5-mm trocar was placed 4 cm below the right costal margin along the midclavicular line as the main operating ports, facilitating the surgeon’s left-handed operation with smooth suturing. An additional 5-mm trocar was placed below the right axillary front line as the assistant operating port to facilitate surgical maneuvers. Intraoperatively, the ampulla of the gallbladder was fortuitously revealed to form a dense adhesion to the duodenum (Figure 2A). Then, we innovatively adopted an artery-first strategy, the cystic artery was clamped and disconnected with caution, fully exposing Calot’s triangle, with evident dilation of the common bile duct (Figure 2B). After occluding the cystic duct, a longitudinal incision was made on the common bile duct, from which stagnant bile overflowed (Figure 2C). Through the bile duct incision, under the guidance of a 5 mm single-use choledochoscope (INNOVEX), an endoscopic basket was used to retrieve the common bile duct stones (Figures 2D,E). After the choledochoscope was performed to inspect the common bile duct up to the papilla of Vater to confirm good function and ensure that no stone remains in the intrahepatic bile ducts, we deemed the placement of a stent unnecessary, the whole layer of the CBD wall was sutured continuously and bidirectionally using 4–0 absorbable monofilament sutures (PDS II, 4–0) (Figure 2F), with a margin of approximately 1.0 mm and a needle pitch of approximately 1.5 mm. The gallbladder was then resected in an antegrade manner, and two drainage tubes were placed above the bile duct incision and at the foramen of Winslow, respectively. The operative time was 135 min, with an estimated blood loss of 20 milliliters. The following day, 10 milliliters of pale yellow, clear fluid were drained, and the drains were subsequently removed. Postoperatively, the patient continued to receive intramuscular injections of 20 mg progesterone injection once daily for 2–3 consecutive days. During the perioperative period, fetal heart monitoring showed no abnormalities, meanwhile, bilirubin and transaminase levels returned to normal. There were no surgical complications, and the patient was discharged on the third postoperative day and recovered well with a marked improvement in jaundice. A follow-up 1 month later revealed no residual common bile duct stones and no abnormalities in fetal development.

## 3 Discussion

It is reported that cholelithiasis complicating pregnancy is the second most common non-obstetric surgical emergency (9), and complicates between 0.05–0.8% of pregnancies (10), whose management continues to be a significant challenge for both general surgeons and obstetricians. Bile sludge during pregnancy is common and usually asymptomatic. Still, its presence in early pregnancy is associated with a high risk of developing gallstones (11), which is related to hormonal changes during pregnancy that lead to increased secretion of cholesterol in bile, reduced enterohepatic circulation, decreased percentage of chenodeoxycholic acid, and bile stasis (12). The management of choledocholithiasis complicated by gallstones during pregnancy presents an intractable clinical issue, with literature reporting that conservative management of gallstone disease during pregnancy significantly increases the rate of maternal readmission (13). Furthermore, acute cholangitis caused by common bile duct stones significantly increases the risk of pregnancy complications (14). Experts have stated that even asymptomatic common bile duct stones require timely intervention to prevent complications (15). Compared with pregnancies without surgery, non-obstetric surgery during pregnancy is not associated with an increased incidence of adverse reproductive outcomes related to specific types of anesthesia or surgery (16). Therefore, surgical treatment should be actively pursued for symptomatic patients regardless of the stage of pregnancy, as the progression of the disease itself or the failure of conservative treatment, leading to premature birth and miscarriage, poses a greater risk than the stress and complications associated with surgery (17). Here, we focus on discussing the minimally invasive treatment methods for choledocholithiasis complicated by gallstones during pregnancy.

Since the early 1990s, LC has been widely recognized as the preferred method for the treatment of gallstone disease, but there is currently no consensus on the treatment of common bile duct stones (18). Heretofore, in the therapeutic strategy for concomitant gallstones and common bile duct stones, the most commonly adopted approaches are either the staged performance of ERCP combined with LC or the simultaneous execution of LC with LCBDE. Current reports on the treatment of gallstone disease during pregnancy mainly focus on the former, while there are few reports on laparoscopic surgery cases that complete LC and LCBDE in the same period (14). However, studies have shown that delayed removal of biliary tract stones during pregnancy is associated with a recurrence of symptoms postpartum (13), and ERCP often requires a second surgery to remove the gallbladder, which undoubtedly increases the duration that the pregnant woman carries the stones in the gallbladder and may adversely affect the fetus and prognosis. For patients with previous surgical procedures on the gastrointestinal reconstruction, endoscopy often cannot reach the second portion of the duodenum, thereby increasing the risk of failure for ERCP (3). Additionally, high incidence rates of post-ERCP complications such as pancreatitis, bleeding, and duodenal perforation (19), along with the potential teratogenic effects of ionizing radiation on the fetus during the procedure, including embryonic death, fetal growth restriction, microcephaly, tumors, and long-term cognitive impairments (20), all contribute to the limitations on the use of ERCP during pregnancy. In comparison, the advantages of the single-session procedure of laparoscopic treatment have become increasingly apparent, and existing literature supports that laparoscopic surgery at any stage of pregnancy is safe and feasible for both the mother and fetus, without adversely affecting pregnancy outcomes (21).

Despite this, the application of laparoscopic technology during pregnancy was once considered an absolute contraindication. Some surgeons are concerned that the intra-abdominal pressure caused by CO2 pneumoperitoneum may lead to fetal hypoperfusion during surgery, thereby increasing the risk of fetal malformation, miscarriage, or preterm birth. However, studies by Cox (22) and Lansten (23) indicate that compared to traditional open surgery, laparoscopic surgery has a lower incidence of postoperative complications, with no statistically significant difference in the rates of fetal loss and preterm birth. This further supports the safety and efficacy of using laparoscopic techniques during pregnancy. Moreover, in the last few years, the introduction of the IoT concept within surgical practice has become one of the most revolutionizing breakthroughs in this field (6). Artificial intelligence (AI), three-dimensional visualization technology, and augmented reality (AR) navigation systems supported by IoT have also provided strong guarantees for the safety and precision of surgical procedures during pregnancy. During the anesthetic management of laparoscopic surgery, considering the special physiological characteristics of pregnant women and fetuses, anesthesia drugs with low lipid solubility and rapid metabolism should be preferentially selected to reduce the risk of adverse effects on the fetus due to drug transfer across the placenta. The potential adverse effects of high pneumoperitoneum pressure or prolonged insufflation should not be overlooked. For surgeries during the second trimester of pregnancy, it is recommended to maintain the pneumoperitoneum pressure between 8 and 12 mmHg to avoid the potential impact of high pneumoperitoneum pressure on uterine blood perfusion, ensuring normal placental function and fetal oxygen supply. In addition, special attention should be paid to the regulation of end-tidal carbon dioxide pressure (EtCO₂), maintaining it between 30 and 35 mmHg to avoid hypercapnia and fetal acidosis (24). By reasonably controlling the pneumoperitoneum pressure and anesthetic parameters, the safety and effectiveness of laparoscopic surgery during pregnancy can be further optimized, thereby reducing the adverse physiological impacts on the mother and fetus.

With the advancement of modern laparoscopic technology, the unique advantages of the single-session procedure of laparoscopic treatment have been proven, as it can address two distinct pathological conditions in a single surgery while avoiding irreversible damage to the sphincter of Oddi and associated adverse events. Its efficiency, safety, and convenience have led to widespread patient acceptance (3). Some researchers have reported that the clearance rate for common bile duct stones treated with LCBDE ranges between 94 and 98%, and it has the advantage of low complication and mortality rates (25). However, regarding the treatment strategy for managing common bile duct stones during pregnancy, a clear and strong agreement has not yet been reached. Only a few cases have shown that the surgery can be performed safely without increasing the risk to pregnant women or fetuses when following recognized management guidelines (Supplementary Table 1). Therefore, there is an urgent need for more case studies to prove its safety and feasibility. Generally, LCBDE includes two approaches: the transcystic duct method and the common bile duct incision method. The choice should take into account the location, size, number, and anatomical structure of the bile duct stones. Generally, the laparoscopic transcystic duct approach is less invasive, has a high clearance rate, and a low risk of bile leakage (26). However, for cases with significantly dilated CBD, large or multiple stones, impacted stones, and intrahepatic stones, as well as a small or tortuous cystic duct, a common bile duct incision is recommended (27). The patient presented with initial symptoms of right upper quadrant abdominal pain accompanied by obstructive jaundice. Combined with abdominal US and MRCP, the examination suggested gallstones with cholecystitis, dilated intra-and extrahepatic bile ducts, and an impacted stone in the lower segment of the common bile duct, measuring approximately 12.8 mm in diameter. Further assessment is required to determine the functionality of the major duodenal papilla. CBD exploration and T-tube drainage are the primary laparoscopic surgical methods for clearing common bile duct stones (4). At present, the necessity of T-tube placement during pregnancy remains controversial. Recent studies have indicated that primary closure of the common bile duct (PCCBD) after LCBDE is a safe and effective alternative to T-tube drainage for the treatment of common bile duct stones (28), this approach does not lead to an increased incidence of biliary strictures, bile leaks, postoperative bleeding, and peritonitis, while also reducing operative time and hospital stay, and enhancing the quality of life for patients (29). Although the T-tube provides convenient percutaneous access for cholangiography and the extraction of residual stones (30), the incidence of complications associated with long-term T-tube placement is as high as 4–16.4% (31). Existing studies have indicated that T-tubes are associated with specific complications, such as cholangitis and bile leakage that may occur upon removal (32), which are particularly detrimental to pregnant women and fetuses. These complications can trigger systemic inflammatory responses or infections in the pregnant woman, causing persistent stimulation to the fetus in the womb, inducing uterine contractions, and increasing the risk of miscarriage or preterm birth. This has the potential to severely impact the prognosis for both mother and fetus.

Traditionally, it is believed that surgery in early pregnancy increases the risk of miscarriage, while surgery in late pregnancy raises the risk of preterm birth, with the lowest risk associated with surgery during the second trimester (33). However, Tolcher et al. (34) have challenged this conclusion by scrutinizing a vast array of literature, and through extensive data analysis, they argue that surgery during the first trimester and late pregnancy does not significantly increase the risk of miscarriage or preterm birth, although they still recommend performing surgery during the second trimester. However, it should be noted that during the later stages of pregnancy, the susceptibility of the uterus to surgical procedures, infection, or hypoxia significantly increases, necessitating vigilant monitoring of uterine activity both intraoperatively and postoperatively to prevent potentially adverse effects on uterine blood perfusion, placental function, and fetal oxygenation. Moreover, for pregnant women beyond 23 weeks of gestation, the supine position during surgery may lead to inferior vena cava compression by the enlarged uterus, resulting in supine hypotensive syndrome, so it is recommended to adopt the left lateral decubitus position or elevate the right hip to mitigate the risk of venous compression and hypotension (35). In this case, the patient was in the second trimester of pregnancy, which is considered a relatively appropriate and safe time for surgery. Intraoperatively, we innovatively employed the “artery-first approach.” This involves dissecting and clamping the cystic artery first during the anatomical dissection of Calot’s triangle, then transecting the cystic duct after the confluence of the cystic duct and the common bile duct has been fully exposed. This not only clarifies the anatomical structures but effectively reduces the risk of biliary injury. However, given its technical complexity, this approach is recommended to be performed by surgeons with extensive experience in laparoscopic surgery. In addition, after confirming complete stone removal and good functionality of the major duodenal papilla through cholangioscopic exploration, we opted for T-tube-free drainage and proceeded directly with the primary suture of the common bile duct. This surgical approach significantly reduced the risks of accidental miscarriage and other related complications that might arise from the indwelling T-tube. We emphasize that timely surgical intervention not only reduced the patient’s readmission rate but also effectively alleviated symptoms, and the collaborative management by a multidisciplinary team further optimized the treatment outcomes for such cases. Although there are currently limited case reports on pregnant women undergoing T-tube-free LCBDE, existing studies have supported this procedure as a safe alternative to ERCP (36, 37). This case further confirms the feasibility and safety of performing a concurrent T-tube-free laparoscopic approach combined with cholangioscopy for common bile duct exploration, stone extraction, and cholecystectomy.

In summary, management of cholelithiasis with choledocholithiasis must be conducted appropriately, a delay in the diagnosis of this pathological condition can increase morbidity and mortality (3). When pregnant women with common bile duct stones present with obstructive jaundice and abdominal pain, surgery should be intervened early, even in the absence of fever, to prevent the onset of shock and mental symptoms. This can effectively halt the further progression of the disease and safeguard the health of both mother and child. For patients undergoing laparoscopic cholecystectomy and CBD exploration with stone extraction followed by primary suture of the CBD, as long as it is confirmed that there are no stones or any other causes of obstruction before the initial closure, and the function of the major duodenal papilla is good, this surgical approach is ideal and safe for patients with simultaneous CBD stones and gallstones during pregnancy. Of course, further studies are needed to substantiate this.

## 4 Conclusion

To sum up, we report a case of a patient in the second trimester of pregnancy with concurrent common bile duct stones and gallstones, who successfully underwent simultaneous T-tube-free laparoscopic cholecystectomy and common bile duct exploration. This approach avoids exposing the developing fetus to ionizing radiation and complications associated with T-tube placement and simultaneously cures common bile duct stones and gallstones during pregnancy. The early involvement of the obstetric team, preoperative and postoperative fetal monitoring, and reasonable management of anesthetics and uterine relaxants make LCBDE during pregnancy seem to be a technically feasible and safe procedure, and it is a viable alternative for the management of concurrent common bile duct stones and gallstones during pregnancy. Infant mortality rate and maternal mortality rate are important indicators of the level of healthcare in a country or region. As surgeons, we should collaborate closely with obstetricians to concentrate on pregnant women suffering from surgical diseases. Through interdisciplinary cooperation and by taking into account the individual circumstances and pregnancy characteristics of the expectant mothers, we can provide precise medical services to reduce the unnecessary risks of preterm birth and miscarriage caused by surgical diseases.

## Data Availability

The original contributions presented in the study are included in the article/Supplementary material, further inquiries can be directed to the corresponding author.
