# Supplementary material for: Common bile duct exploration with choledochotomy and primary repair during pregnancy: Case Report
Source: Front Med (Lausanne). 2025 Apr 15;12:1559568. doi: 10.3389/fmed.2025.1559568 (PMC12037557; doi:10.3389/fmed.2025.1559568)
Supplement: Supplementary file 1 [file Table_1.docx]

Supplementary Material

# Supplementary Tables

**Supplementary Table 1.** Case series on laparoscopic exploration of common bile duct during pregnancy.

| Study | N | Trimester | | | Spontaneous Abortion | Preterm | Followed to Delivery | Technique for LCBDE* | |
| --- | --- | --- | --- | --- | --- | --- | --- | --- | --- |
|  |  | Ⅰ  (0–13 weeks) | Ⅱ  (14–26 weeks) | Ⅲ  (27–40 weeks) |  |  |  | Transductal | Transcystic |
| Víctor et al.[35] | 8 | 3 | 3 | 2 | 0 | 0 | 7^▲^ | 3 | 5 |
| Liberman et al.[37] | 2 | 1 | 1 | 0 | 0 | 0 | 2 | 0 | 2 |
| Zhang et al.[38] | 4 | 0 | 4 | 0 | 0 | 0 | 4 | 0 | 4 |
| Tuech et al.[39] | 1 | 0 | 1 | 0 | 0 | 0 | 1 | 1 | 0 |
| Qiu et al.[40] | 1 | 1 | 0 | 0 | 0 | 0 | 1 | 0 | 1 |
| LCBDE*=Laparoscopic common bile duct exploration;^▲^One patient not followed to delivery. | | | | | | | | | |

**Supplementary Table 2.**

| Abbreviations | |
| --- | --- |
| US | Ultrasonography |
| MRCP | Magnetic Resonance Cholangiopancreatography |
| CBD | Common Bile Duct |
| LC | Laparoscopic Cholecystectomy |
| LCBDE | Laparoscopic Common Bile Duct Exploration |
| PreERCP | Preoperative Endoscopic Retrograde Cholangiopancreatography |
| IntraERCP | Intraoperative ERCP |
| PostERCP | Postoperative ERCP |
| G2P1 | Gestation 2, Production 1 |
| BP | Blood Pressure |
| RR | Respiratory Rate |
| PR | Pulse Rate |
| ALT | Alanine Aminotransferase |
| AST | Aspartate Aminotransferase |
| CBC | Complete Blood Count |
| MDT | Multidisciplinary Team |
| PCCBD | Primary Closure of the Common Bile Duct |
